# A novel point-of-care test for cirrhosis based on dimeric to monomeric IgA ratio in blood: a pilot cohort study

**DOI:** 10.1097/HC9.0000000000000106

**Published:** 2023-03-30

**Authors:** Jessica Howell, Huy Van, Minh D. Pham, Rohit Sawhney, Fan Li, Purnima Bhat, John Lubel, William Kemp, Steven Bloom, Avik Majumdar, Geoff McCaughan, Timothy Spelman, Joseph S. Doyle, Margaret Hellard, Kumar Visvanathan, Alexander Thompson, David Anderson

**Affiliations:** 1 Burnet Institute, Melbourne, Victoria, Australia; 2 St Vincent’s Hospital and University of Melbourne, Melbourne, Victoria, Australia; 3 School of Public Health and Preventive Medicine, Monash University, Melbourne, Victoria, Australia; 4 Eastern Health, Box Hill, Victoria, Australia; 5 Canberra Hospital, Canberra, Australia; 6 Department of Gastroenterology, The Alfred Hospital and Monash Central Clinical School, Melbourne, Victoria, Australia; 7 Royal Prince Alfred Hospital, Sydney, New South Wales, Australia; 8 Centenary Institute, Sydney, New South Wales, Australia; 9 Department of Infectious Diseases, The Alfred and Monash University, Melbourne, Victoria, Australia

## Abstract

Dimeric IgA to monomeric IgA ratio (dIgA ratio) is a biomarker of gut mucosal leakage in liver cirrhosis. We evaluated the diagnostic performance of a novel point-of-care (POC) dIgA ratio test for cirrhosis.

**Methods:**

Plasma samples from people with chronic liver disease were analyzed using the BioPoint POC dIgA ratio antigen immunoassay lateral flow test. Cirrhosis was defined by Fibroscan>12.5 kPa, clinical evidence of cirrhosis or liver histopathology. POC dIgA test diagnostic accuracy was determined in a test cohort using receiver operating characteristic curve analysis; optimal cutoffs for sensitivity and specificity were then applied to a validation cohort.

**Results:**

A total of 1478 plasma samples from 866 patients with chronic liver disease were included (test cohort n = 260, validation cohort n = 606). In all, 32% had cirrhosis; 44% Child-Pugh A, 26% Child-Pugh B, and 29% Child-Pugh C. Median POC dIgA ratio was higher in cirrhosis (0.9) compared with no cirrhosis (0.4, *p* < 0.001), and in Child-Pugh class B/C compared with A cirrhosis (1.4 Child-Pugh B/C vs. 0.6 Child-Pugh A, *p* < 0.001). POC dIgA ratio test had good diagnostic accuracy for liver cirrhosis in the test cohort (area under the receiver operating characteristic curve=0.80); a dIgA ratio cutoff of 0.6 had a sensitivity of 74% and specificity of 86%. POC dIgA test accuracy was moderate in the validation cohort (area under the receiver operating characteristic curve=0.75; positive predictive value 64%, negative predictive value 83%). Using a dual cutoff approach, 79% of cirrhosis cases were correctly diagnosed and further testing was avoided in 57%.

**Conclusions:**

POC dIgA ratio test had moderate accuracy for cirrhosis. Further studies evaluating the accuracy of POC dIgA ratio testing for cirrhosis screening are warranted.

## Graphical abstract

**Figure:**
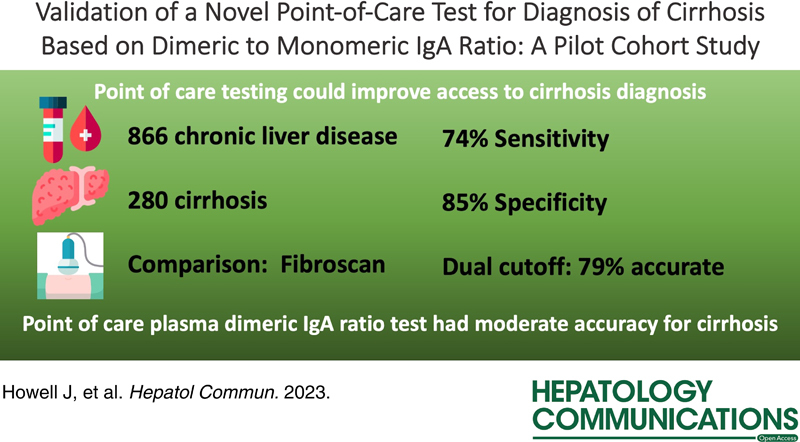

## INTRODUCTION

Cirrhosis is a major cause of morbidity and mortality worldwide^1^,^2^ and both incidence and mortality are steadily increasing, reflecting the global viral hepatitis, alcohol, and fatty liver disease epidemics.^2^,^3^ Without timely diagnosis and treatment of underlying liver disease, people with cirrhosis are at risk of liver decompensation and liver cancer (HCC).^4^ However, most people with early (compensated) liver cirrhosis feel well until liver disease is advanced, at which time it is often too late to prevent life-threatening complications.

Diagnosis of cirrhosis is a critical step in the liver disease management pathway, guiding the choice and duration of pharmacotherapy.^5^–^7^ People with cirrhosis require specialist care, including preventive strategies to reduce the risk of life-threatening complications such as ascites, infections, and variceal bleeding^4^,^8^,^9^ and HCC surveillance.^10^ However, diagnosis of cirrhosis remains a significant barrier to timely treatment of viral hepatitis in low-resource and remote settings, where access to elastography and liver biopsy are limited.^11^,^12^ Blood-based biomarkers of liver fibrosis such as the aspartate aminotransferase (AST) to platelet ratio index (APRI)^13^ have therefore been developed to overcome limited access to liver biopsy and elastography. However, these biomarker algorithms still require specialized blood-based assays performed in centralized laboratory services, presenting logistical, cost, and resource challenges to optimal liver disease management.^11^,^14^ Moreover, access to diagnostic modalities for portal hypertension among people with cirrhosis is extremely limited in low-resource settings. Rapid blood-based point-of-care (POC) tests have the potential to overcome many of the barriers to timely diagnosis of liver cirrhosis in low-resource settings: they provide a rapid on-the-spot diagnosis to guide management, are affordable and easy to use, and can be used anywhere to facilitating decentralized models of liver disease care.^15^ However, there is currently no blood-based POC test for cirrhosis.

In the healthy state, dimeric and monomeric IgA are secreted into the gut lumen and dimeric IgA does not readily cross the mucosal barrier into the splanchnic circulation.^8^ However, the development of portal hypertension in liver cirrhosis leads to increased gut permeability, exacerbated by low oncotic pressure, increased inflammation, and bacterial translocation.^16^ This leads to greater passage of dimeric IgA into the bloodstream across the gut mucosal barrier,^8^ increasing the ratio of dimeric to monomeric IgA in blood.^17^,^18^ Several small studies have shown that dimeric to monomeric IgA ratio (dIgA ratio) is increased in states of gut leakage,^17^,^18^ such as in HIV infection^19^ and alcohol-associated liver cirrhosis.^20^

We, therefore, developed a prototype POC antigen immunoassay lateral flow test for quantification of dIgA using specific antibodies to the dimeric form of the antibody molecule. The POC dIgA test provides a visual numerical result in 30 minutes from a droplet of plasma, read by the small Axxin handheld lateral flow reader. This test can also be adapted to measure the dIgA ratio in a single droplet of whole blood.^21^

Our study describes the development of an in-house prototype lateral flow assay that measures 3 IgA subclasses (secretory IgA, dimeric IgA, and total IgA). We evaluated the association between plasma IgA subclasses and liver cirrhosis in a large cohort of people with chronic liver disease and healthy controls and determined the diagnostic accuracy of a novel lateral flow POC test for liver cirrhosis based on dimeric to monomeric IgA subclass ratio (dIgA ratio) measured in plasma.

## METHODS

### Study design and population

This cross-sectional retrospective cohort study conducted in 2020 included plasma samples and demographic and clinical data from adults over 18 years with chronic liver disease and healthy controls sourced from several Australian clinical trial cohorts and biobanks, including CATCH study,^22^ STOP study,^23^ TAP study,^24^ Austin Hospital cirrhosis study, Royal Prince Alfred Hospital Liver Biobank, St Vincent’s Hospital Liver Biobank and Burnet Institute COVID-19 study (Supplemental Table S1, http://links.lww.com/HC9/A225). Samples and data were collected during the preceding 5 years (Austin cirrhosis cohort collected within 10 y) of the study. All participants were consented in accordance with institutional ethics requirements and agreed to contribute clinical samples and data to this study. The study was conducted in accordance with the Declaration of Helsinki and was approved by the St Vincent's Hospital institutional ethics committee. Age less than 18 years was the only exclusion criterion.

All deidentified samples were collected in accordance with the study and Biobank protocols. Matched deidentified demographic and clinical data were linked by study number to the plasma samples. All samples were stored at −80°C in temperature-monitored freezers. Cirrhosis was defined using international disease-specific liver stiffness criteria in kPa^5^ (Supplemental Methods Table S2, http://links.lww.com/HC9/A225) and/or liver biopsy histopathology per study and biobank protocols. Portal hypertension was defined as the presence of esophageal or gastric varices and/or portal hypertensive gastropathy on endoscopy; or a platelet count <150×10^9^/L and splenomegaly and features of portal hypertension on liver ultrasound; or clinical evidence of ascites.

First, we used samples from a set of 20 patients with cirrhosis and 17 healthy controls to determine the relationship between IgA subclasses and cirrhosis status. Then, from the whole cohort, we selected a random test cohort of 260 individuals (30% of the total cohort) to explore the association between cirrhosis and dimeric IgA ratio and develop test cutoffs for cirrhosis using STATA (StataCorp Inc.). Due to the varying distribution of liver diseases within the study cohorts from which samples were derived for this study, the selection was weighted to ensure ∼1:2 ratio of hepatitis C (HCV) and hepatitis B (HBV) patients, respectively in the test cohort. Individuals that were not selected for the test cohort were assigned to the validation cohort.

### Measurement of IgA subclasses by lateral flow assay

Secretory IgA, dimeric IgA, and total IgA in plasma samples from healthy volunteers and patients with cirrhosis were measured using a noncommercial novel prototype rapid POC lateral flow assay that was developed in-house. Antisecretory monoclonal antibody, recombinant chimeric secretory component, and protein L were immobilized on a nitrocellulose membrane to capture secretory IgA, dimeric IgA, and total IgA from whole blood or plasma, respectively. The lateral flow strip comprises a reference line that binds total IgA (also providing a measure of IgA deficiency as a clinical control), and a test line that binds dIgA, the relative intensity of which is used as a semiquantitative measure of the proportion of total IgA (usually only 10%). The IgA subclasses were detected with anti-human IgA conjugated to colloid gold and the amount of the IgA was measured by an Axxin reader, which was calibrated to output the ratio of monomeric to dimeric IgA numerically.

Plasma samples were thawed at ambient temperature, centrifuged at 5000*g* for 5 minutes, and transferred to clean Eppendorf tubes. A volume of 5 μL of plasma was added to well A of the POC dIgA device, followed by 1 drop of running buffer. After waiting 10 minutes, 4 drops of running buffer were added to well B. Results were interpreted after 20 minutes in the Axxin handheld reader to obtain a semiquantitative result. dIgA ratio was calculated using the Axxin handheld reader and recorded by a scientist blinded to the clinical data and cirrhosis status of the participant from which the blood sample had been derived.

### Calculation of APRI and Fibrosis-4 (FIB-4)

APRI was calculated using the formula:


$$\mathrm{APRI}\;=\;\frac{\mathrm{AST}\;(\mathrm{IU}/L)/\mathrm{AST}\;\mathrm{upper}\;\mathrm{limit}\;\mathrm{normal}\;(\mathrm{IU}/L)}{\mathrm{Platelet}\;\mathrm{count}\;{(\mathrm{10}}^{9}/L)}\;\times \;100.$$

An APRI cutoff of >1 was used to diagnose cirrhosis.

FIB-4 was calculated using the formula:


$$\mathrm{FIB}-4\;=\;\frac{\mathrm{Age}\left(y\right)\;\times \;\mathrm{AST}\;\left(\mathrm{IU}/L\right)}{\mathrm{Platelet}\;\mathrm{count}\;{(\mathrm{10}}^{9}/L)\;\times \;\sqrt{\mathrm{ALT}\;(\mathrm{IU}/L})}.$$

A lower cutoff of <1.45 was used to exclude cirrhosis, whereas a higher cutoff of 3.25 was used to diagnose cirrhosis.

### Statistical analysis

The distribution of demographic and clinical variables overall and within the test and validation cohorts were described using number (proportion) for categorical data and mean±SD or median [interquartile range (IQR)] for continuous data.

Associations between the dIgA ratio and the presence and severity of liver cirrhosis were determined in the test cohort. Comparisons of the dIgA ratio between individuals with and without cirrhosis, and between individuals with early-stage and late-stage cirrhosis (Child-Pugh class A compared with B/C class) were performed using the Wilcoxon rank-sum test. Comparisons in median dIgA ratio levels were made between those with and without portal hypertension, hepatic encephalopathy, and ascites, respectively using the Wilcoxon rank-sum test. Regression analysis was used to determine the correlation between the dIgA ratio and serum albumin and bilirubin as markers of liver function.

To measure test performance, a logistic regression model was developed and the area under the receiver operating characteristic curve (AUROC) of the POC dIgA ratio for cirrhosis was determined in the test cohort. A Youden index was calculated and the sensitivity and specificity of different cutoffs of the dIgA ratio were determined (test cohort; Supplemental Results Table S5, http://links.lww.com/HC9/A225). Based on these results, a dIgA ratio cutoff was selected to optimize sensitivity and specificity and applied to the validation cohort to determine the diagnostic performance of the dIgA ratio for cirrhosis status by AUROC. Sensitivity, specificity, positive predictive value (PPV), and negative predictive value (NPV) were calculated by the confusion matrix. We additionally explored the impact of restricting the use of the POC dIgA ratio to those aged>30 years on accuracy for cirrhosis diagnosis in the validation cohort.

A dual cutoff method for optimizing the accuracy of the dIgA ratio test for the diagnosis of cirrhosis was investigated in the validation cohort. Two dIgA ratio cutoffs were selected to optimize sensitivity (“rule-out”) and specificity (“rule-in”), drawn from sensitivity and specificity values previously generated using different dIgA cutoffs (Supplemental Results Table S5, http://links.lww.com/HC9/A225).

The diagnostic accuracy of the dIgA ratio for diagnosis of portal hypertension was determined in the whole cohort to increase the number of individuals with portal hypertension as the outcome of interest. Among the subset of patients who had endoscopy data available, the accuracy of the dIgA ratio for diagnosis of esophageal varices was also determined.

A 2-sided *p*-value of 0.05 was set as the threshold for statistical significance. All analyses were performed using STATA, v14 (StataCorp Inc.).

## RESULTS

### IgA subclass comparison

**FIGURE 1:**
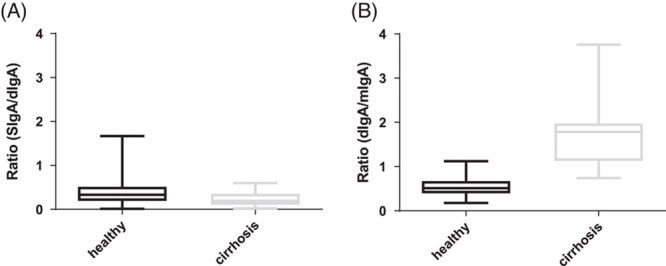
(A) Secretory IgA to dimeric IgA ratio in patients with cirrhosis (n = 20) compared with healthy controls (n = 17). (B) Dimeric IgA to monomeric IgA ratio in patients with cirrhosis (n = 20) compared with healthy controls (n = 17).

Secretory IgA, dimeric IgA, and total IgA levels from 17 healthy individuals and 20 patients with cirrhosis were measured by lateral flow assay. The mean ratio of secretory IgA to dimeric IgA levels in plasma were not significantly different between patients with cirrhosis (0.49) and healthy controls (0.29, *p* = 0.04; Figure 1A). However, the mean ratio of dimeric IgA to monomeric IgA levels were significantly higher in patients with cirrhosis (1.72) than in healthy individuals (0.54, *p* < 0.001; Figure 1B).

Tests to determine the normal reference range, interobserver variability, and coefficient of variance for the POC dIgA test are shown in Supplemental Results and Supplemental Table S3 (http://links.lww.com/HC9/A225). POC dIgA results were congruent across duplicate samples. A comparison between the dIgA ratio measured in plasma compared with whole blood is shown in Supplemental Table S4 (http://links.lww.com/HC9/A225).

### Study cohort

Eight hundred sixty-six individuals were included in the study; 260 individuals provided plasma samples for the test cohort and 606 individuals provided 1218 plasma samples for the validation cohort. The distribution of clinical and demographic variables is outlined in Table 1. Most were male (60%) with a mean age of 48 years; 60% had HBV infection and 20% had HCV. Overall, 32% had liver cirrhosis and 15% had clinical evidence of portal hypertension. There was no significant difference in the distribution of clinical or demographic variables between the test and validation cohorts (Table 1).

**TABLE 1:** Distribution of clinical and demographic factors in the test and validation cohorts (N = 866)

	n (%)		
Variables	Test cohort (n = 260)	Validation cohort (n = 606)	Total (N = 866)
Male	138 (53)	346 (57)	484 (56)
Female	104 (40)	223 (37)	327 (38)
Missing	18 (7)	37 (6)	55 (6)
Age (mean±SD) (y)	47.5±14.2	48.0±13.4	47.8±13.7
Healthy controls	10 (4)	27 (4)	37 (4)
Hepatitis B	153 (59)	359 (59)	512 (59)
Hepatitis C	52 (20)	123 (20)	175 (20)
MAFLD (n = 268)^a^	13 (5)	38 (6)	51 (6)
ALD (n = 268)^a^	31 (37)	59 (32)	90 (34)
Other liver disease	18 (7)	41 (7)	59 (7)
Cirrhosis	88 (34)	192 (32)	280 (32)
Child-Pugh class			
A	29 (33)	62 (32)	91 (33)
B	15 (17)	36 (19)	51 (18)
C	20 (23)	39 (20)	59 (21)
Missing	24 (27)	55 (29)	79 (28)
Fibroscan (n = 507) [median (IQR)] (kPa)^a^	4.8 (4.0, 6.1)	4.8 (3.9, 6.0)	4.8 (3.9, 6.0)
Portal hypertension	39 (15)	80 (13)	119 (14)
Active HCC	22 (10)	50 (10)	118 (14)
dIgA ratio [median (IQR)]	0.44 (0.28, 0.76)	0.45 (0.30, 0.69)	0.47 (0.31, 0.75)

^a^ MAFLD and ALD status and Fibroscan data only available for a subset of patients.

Abbreviations: ALD, alcohol-associated liver disease; dIgA, dimeric IgA to monomeric IgA ratio; IQR, interquartile range; MAFLD, metabolic-associated fatty liver disease.

### Association between dimeric IgA ratio and liver cirrhosis

**FIGURE 2:**
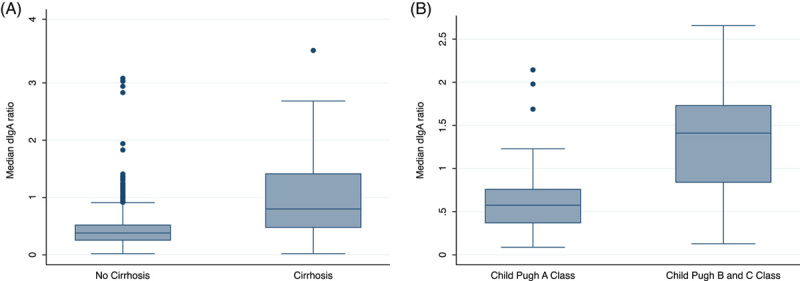
(A) Dimeric IgA ratio in patients with cirrhosis (n = 88) compared with those without cirrhosis (n = 200). Median dIgA ratio was 0.86 (IQR = 0.60, 1.44) in individuals with cirrhosis compared with 0.36 (IQR = 0.25, 0.49) in those with no cirrhosis (*p* < 0.001). (B) Dimeric IgA ratio in patients with Child-Pugh class A cirrhosis (n = 53) compared with class B or C cirrhosis (n = 35). Median dIgA ratio was 1.41 (IQR = 0.84, 1.73) in those with Child-Pugh B and C decompensated cirrhosis compared with 0.58 (IQR = 0.37, 0.76) in those with Child-Pugh A stage compensated cirrhosis (*p* < 0.001). Abbreviations: dIgA, dimeric IgA to monomeric IgA ratio; IQR, interquartile range.

The relationship between the dIgA ratio and cirrhosis status was determined using samples from the test cohort (n = 260). The median dIgA ratio was significantly higher in plasma samples from people with liver cirrhosis [0.86 (0.60, 1.44)] compared with no cirrhosis [0.36 (0.25, 0.49), rank sum *p* < 0.001] (Figure 1). The median dIgA ratio was also higher in patients with compensated Child-Pugh A cirrhosis (0.62; IQR = 0.39, 0.92) compared with those without cirrhosis (0.38, IQR = 0.26, 0.52, rank sum *p* < 0.001) and higher in patients with decompensated Child-Pugh B and C stage cirrhosis (median dIgA ratio of 1.41; IQR = 0.84, 1.73) compared with compensated Child-Pugh A stage cirrhosis [median dIgA ratio of 0.58 (IQR = 0.37, 0.76), *p* < 0.001, Figure 2]. Median dIgA ratio levels were significantly higher in cirrhosis compared with no cirrhosis irrespective of underlying liver disease, however, dIgA ratio levels were lower in people with HBV compared with HCV and other causes of liver disease (Supplemental methods, Figure S1, http://links.lww.com/HC9/A225).

### Association between dIgA ratio and liver function

**FIGURE 3:**
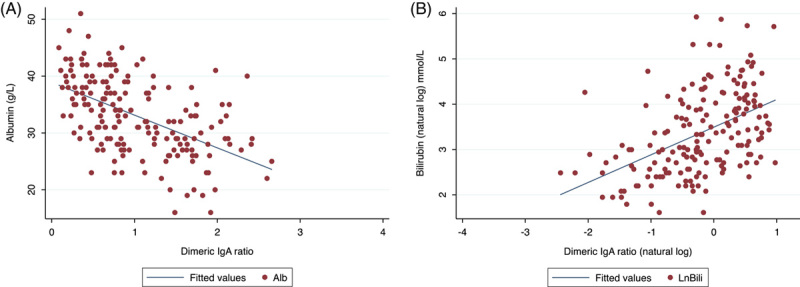
(A) Correlation between albumin and ratio IgA. *p* < 0.001, adjusted *R* ^2^ = 0.29 (n = 181). (B) Correlation between natural log bilirubin and natural log dimeric IgA ratio. *p* < 0.001, adjusted *R* ^2^ = 0.22 (n = 181).

In a subset of 181 participants who had albumin and bilirubin data available (Austin hospital pretransplant cohort, RPA biobank, and CATCH study cohorts, see Supplemental Table S1, http://links.lww.com/HC9/A225), dIgA ratio was associated with markers of liver function: albumin was inversely correlated with dIgA ratio (adjusted *R* ^2^ = 0.3, *p* < 0.001) and bilirubin correlated weakly with dIgA ratio (adjusted *R* ^2^ = 0.1, *p* < 0.001, Figure 3). There was no correlation between alanine aminotransferase levels and the dIgA ratio (*p* = 0.986, adjusted *R* ^2^ = −0.008).

### Association between dIgA ratio and portal hypertension

A total of 809 individuals had portal hypertension data available. The median dIgA ratio was higher in those with portal hypertension [1.14 (0.72, 1.68)] compared with those without [0.39 (0.26, 0.55; *p* < 0.001]. Moreover, individuals with complications of portal hypertension had higher dIgA ratio levels than those who did not, including hepatic encephalopathy [1.4 (0.72, 1.72) compared with 0.43 (0.29, 0.65; *p* < 0.001] and ascites [1.41 (0.81, 1.74) compared with 0.40 (0.27, 0.58), *p* < 0.001]. Within the subset of 688 individuals with gastroscopy report data available, patients with varices had higher dIgA ratio than those without varices [median dIgA ratio=0.77 (0.58, 1.16) compared with 0.39 (0.26, 0.55), *p* < 0.001]. There was a weak inverse correlation between platelet count and dIgA ratio (adjusted *R* ^2^ = −0.18, *p* < 0.001; Figure S2, http://links.lww.com/HC9/A225).

### Diagnostic performance of dimeric IgA ratio for cirrhosis

**FIGURE 4:**
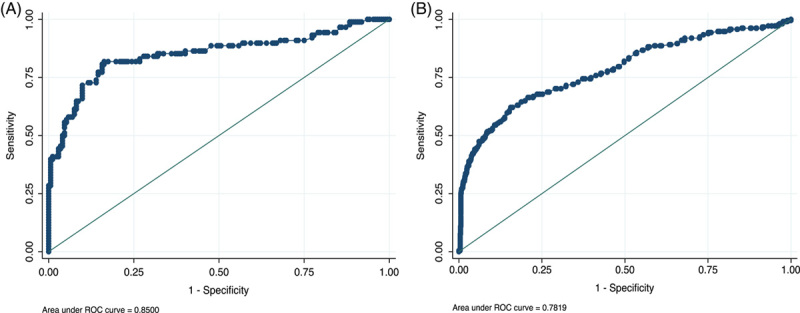
(A) AUROC ratio IgA for cirrhosis, test set (n = 260). *p* < 0.001. AUROC = 0.80 (95% CI: 0.75–0.85). (B) AUROC ratio IgA for cirrhosis, validation set (n = 1218). *p* < 0.001. AUROC = 0.75 (95% CI: 0.72–0.78). Abbreviation: AUROC, area under the receiver operating characteristic curve.

In the test cohort, the diagnostic performance of the POC dIgA ratio for cirrhosis was good, with an AUROC of 0.85 (95% CI: 0.79–0.91, *p* < 0.001) (Figure 4A). A dIgA ratio cutoff of 0.6 was selected to optimize diagnostic performance (cirrhosis prevalence 34%; (Supplemental Table S5, http://links.lww.com/HC9/A225), which yielded a sensitivity of 74%, specificity of 86%, PPV of 73%, and NPV of 84%. POC dIgA ratio had moderate accuracy for cirrhosis in the validation cohort on baseline blood samples, with an AUROC of 0.74 (95% CI: 0.70–0.77) (Figure 4B); sensitivity (64%), specificity (83%), PPV (64%), and NPV (83%). Diagnostic performance was similar in longitudinal repeat plasma samples from individuals in the validation cohort (AUROC = 0.78, 95% CI: 0.74–0.82; sensitivity 62%, specificity 84%, PPV 45%, NPV 91%; Table 2). Data on the stability of POC dIgA ratio test results over time are presented in Supplemental Results (http://links.lww.com/HC9/A225).

**TABLE 2:** Diagnostic performance of POC dimeric IgA test for cirrhosis using a cutoff of 0.6

dIgA ratio	Sensitivity (%)	Specificity (%)	PPV (%)	NPV (%)	AUROC	95% CI	*p*
Test cohort (n = 260)							
>0.6	74	86	73	84	0.80	0.75–0.85	<0.001
Validation cohort: baseline samples (n = 606)							
>0.6	64	83	64	83	0.74	0.70–0.77	<0.001
Validation cohort: longitudinal samples (n = 1218)							
>0.6	62	84	45	91	0.75	0.72–0.78	<0.001
Whole cohort, aged over 30 years (n = 866, 1478 samples)							
>0.6	67	84	68	83	0.81	0.77–0.84	<0.001
APRI (n = 434)							
>1.0	55	99	92	90	0.93	0.89–0.96	<0.001
FIB-4 (n = 434)							
<1.45	89	86	59	97	0.87	0.83–0.91	<0.001
>3.25	66	99	98	93	0.83	0.78–0.88	<0.001

Abbreviations: APRI, aspartate aminotransferase to platelet ratio index; AUROC, area under the receiver operating characteristic curve; dIgA, dimeric IgA to monomeric IgA ratio; FIB-4, Fibrosis-4; NPV, negative predictive value; PPV, positive predictive value; POC, point-of-care.

Age is a key risk for liver fibrosis and liver cirrhosis is uncommon under 30 years of age.^25^ Therefore, we explored the diagnostic accuracy of the POC dig A ratio exclusively in those aged over 30 years (n = 57) (Table 2). This approach improved the PPV slightly to 68%, however, missed 4 (7%) aged below 30 who had cirrhosis.

Using the whole cohort, we also explored differences in the diagnostic performance of the POC dIgA ratio test within subgroups, stratifying by HBV and HCV status, biological sex, and clinically compensated cirrhosis (Table 3). Among 512 individuals (whole cohort) with HBV, the POC dIgA ratio was less accurate compared with those with other liver diseases, with AUROC of 0.69 (95% CI: 0.59–0.78), the sensitivity of 51%, specificity of 84%, PPV 23%, and NPV of 95%. Only 0.7%^7^ of people with HBV had advanced Child-Pugh B or C stage cirrhosis. The POC dIgA ratio performed less strongly for cirrhosis diagnosis in people with no clinically apparent signs of cirrhosis, with sensitivity of 60% and NPV of 90%.

**TABLE 3:** Diagnostic performance of the POC dIgA ratio test by subgroup

dIgA ratio	Sensitivity (%)	Specificity (%)	PPV (%)	NPV (%)	AUROC	95% CI	*p*
Hepatitis B (n = 508)							
>0.6	53	84	22	95	0.70	0.60–0.80	<0.001
Hepatitis C (n = 149)							
>0.6	74	87	84	78	0.82	0.75–0.89	<0.001
Whole cohort, excluding those with physical signs of cirrhosis^a^ (n = 672)							
>0.6	60	84	47	90	0.74	0.68–0.80	<0.001
Males (n = 484)							
>0.6	68	86	78	79	0.819	0.78–0.86	<0.001
Females (n = 327)							
>0.6	54	82	38	89	0.734	0.65–0.81	<0.001
POC dIgA ratio in whole cohort, excluding those with physical signs of cirrhosis^a^ (n = 672)							
>0.6	60	84	47	90	0.74	0.68–0.80	<0.001
APRI in whole cohort, excluding those with physical signs of cirrhosis							
>1.0	37	99	80	93	0.68	0.61–0.75	<0.001
FIB-4 in whole cohort, excluding those with physical signs of cirrhosis							
>3.25	47	95	95	94	0.73	0.66–0.81	<0.001

^a^ Clinically evident ascites, hepatic encephalopathy, clinical jaundice, and/or peripheral stigmata of cirrhosis or portal hypertension at time of test.

Abbreviations: APRI, aspartate aminotransferase to platelet ratio index; AUROC, area under the receiver operating characteristic curve; dIgA, dimeric IgA to monomeric IgA ratio; FIB-4, Fibrosis-4; NPV, negative predictive value; PPV, positive predictive value; POC, point-of-care.

### Comparison of POC dIgA ratio with APRI and FIB-4 for diagnosis of cirrhosis

**FIGURE 5:**
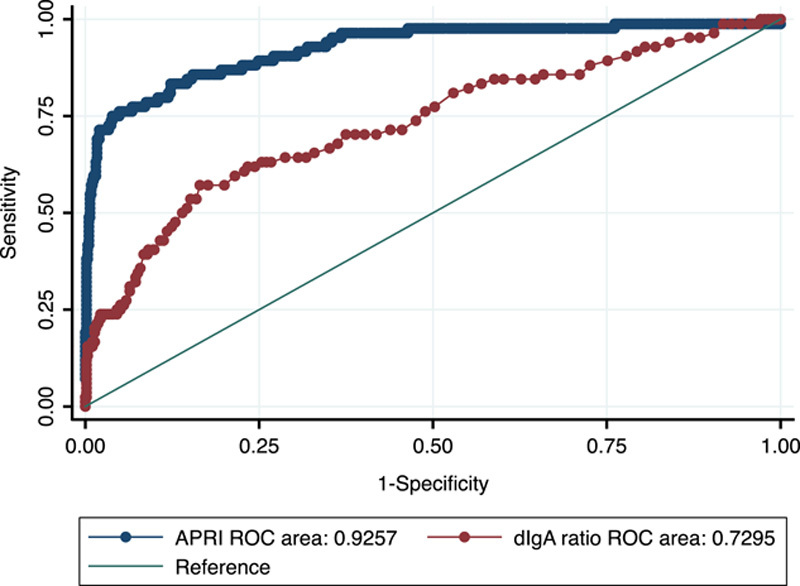
AUROC APRI for cirrhosis (n = 434). AUROC = 0.93 (95% CI: 0.87–0.960, *p* < 0.001). AUROC ratio IgA for cirrhosis (n = 434). AUROC = 0.72 (95% CI: 0.65–0.78, *p* < 0.001). Abbreviations: APRI, aspartate aminotransferase to platelet ratio index; AUROC, area under the receiver operating characteristic curve; dIgA, dimeric IgA to monomeric IgA ratio; ROC, receiver operating characteristic.

APRI and FIB-4 could be calculated in a subset of 434 study participants across the test and validation groups. APRI had excellent discrimination for cirrhosis, with APRI >1 having an AUROC of 0.93 (95% CI: 0.89–0.96) for cirrhosis (Figure 5). APRI>1.0 had a sensitivity of 54%, specificity of 99%, PPV of 89%, and NPV of 94% for cirrhosis (Table 2). FIB-4 had excellent diagnostic accuracy for cirrhosis in the same cohort of 434 individuals, with an AUROC of 0.94 (95% CI: 0.90–0.98). Using the higher cutoff of 3.25 had a sensitivity of 66%, specificity of 99%, and NPV of 93% for cirrhosis (Table 2). In comparison, the dIgA ratio had lower discriminative ability but higher sensitivity than APRI and similar sensitivity to FIB-4 for cirrhosis in the subset of study participants with APRI and FIB-4 data (AUROC = 0.72, 95% CI: 0.65–0.78). In those with clinically silent cirrhosis, both FIB-4 and APRI had lower sensitivity but higher specificity for cirrhosis compared with the POC dIgA ratio test (FIB-4 sensitivity 47%, specificity 95%; APRI sensitivity 37%, specificity 99%, Table 3).

Among the 68 people who had intermediate FIB-4 results, POC dIgA testing using the 0.6 cutoff detected an additional 5 cirrhosis cases and correctly identified 38 (78%) as not having cirrhosis, avoiding further transient elastography.

### Optimizing the diagnostic performance of the POC dIgA ratio test using “rule-in” and “rule-out” dIgA ratio cutoffs

**FIGURE 6:**
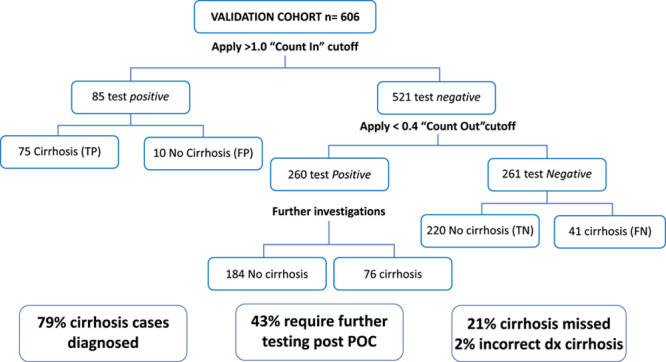
Diagnostic outcome of using a “Count in” dIgA ratio cutoff of >1.0 and a “count out” dIgA ratio cutoff of <0.4 to diagnose cirrhosis (n = 1105). Overall, 75/192 cirrhosis cases were correctly identified by a POC dIgA ratio of >1.0 and 220/414 were correctly identified as having no cirrhosis using a POC dIgA ratio <0.4. In total, 260/606 (43%) with a dIgA ratio between 0.4 and 1.0 would require further investigations such as transient elastography. There were 41 false negatives with cirrhosis missed (21%) and 10 false positives without cirrhosis (2%). Abbreviations: dIgA, dimeric IgA to monomeric IgA ratio; FN, false negative; FP, false positive; POC, point-of-care; TN, true negative; TP, true positive.

Similar to the dual cutoffs employed in FIB-4 calculation,^5^ we explored the impact of using a high sensitivity cutoff of <0.4 to “count out” people with a very low risk of cirrhosis and a high specificity cutoff of 1.0 to “count in” people with a high risk of cirrhosis, thereby restricting the need for further fibrosis investigations to people with an indeterminate POC dIgA ratio between 0.4 and 1.0 (Figure 6). Using this method, we detected 79% of cirrhosis cases if patients with dIgA ratio levels>1 were classified as cirrhosis and those with dIgA ratio between 0.4 and 1.0 went on to have definitive testing for cirrhosis with elastography; 2% were falsely classified as having cirrhosis and 21% of cirrhosis cases were missed (false negatives). Overall, 43% had an indeterminate POC dIgA ratio between 0.4 and 1.0 and would require further investigations such as elastography to diagnose cirrhosis. This dual-cutoff POC dIgA ratio approach was compared favorably with APRI, which had a sensitivity of only 55%, resulting in 46% of cirrhosis cases, or 88 individuals, being missed. In comparison, the use of the FIB-4 dual cutoff approach (classifying those with FIB-4>3.25 as cirrhosis, with further investigations for people with FIB-4 between 1.45 and 3.25) was more accurate than the dual cutoff POC dIgA ratio test approach: 11% of cirrhosis cases were missed, 16% required further fibrosis assessment and the false-positive rate was 1%.

Interestingly, when we used this approach exclusively in those aged over 30 years who have a higher pretest probability of cirrhosis, there was no improvement in diagnostic accuracy and no reduction in the proportion of cirrhosis cases missed (21% missed cirrhosis cases, 2% false-positive cases, further investigations spared in 57%).

Among the 41 cirrhosis cases missed using the dIgA ratio dual cutoff approach, 8 (21%) had portal hypertension and most were Child-Pugh class A (80%). A higher proportion had HBV (29% vs. 10%, *p* = 0.002), a lower proportion had portal hypertension (21% vs. 60%, *p* < 0.001) and there was a trend toward a higher proportion with active HCC at the time of sampling (59% vs. 42%, *p* = 0.09) compared with those where cirrhosis was diagnosed by dIgA ratio. Age, gender, and HCV etiology were otherwise similar between both groups.

APRI score was >1 in 5 (12%) of 41 with cirrhosis missed by dIgA ratio testing.

### Diagnostic accuracy of POC dIgA ratio testing for portal hypertension and varices

**FIGURE 7:**
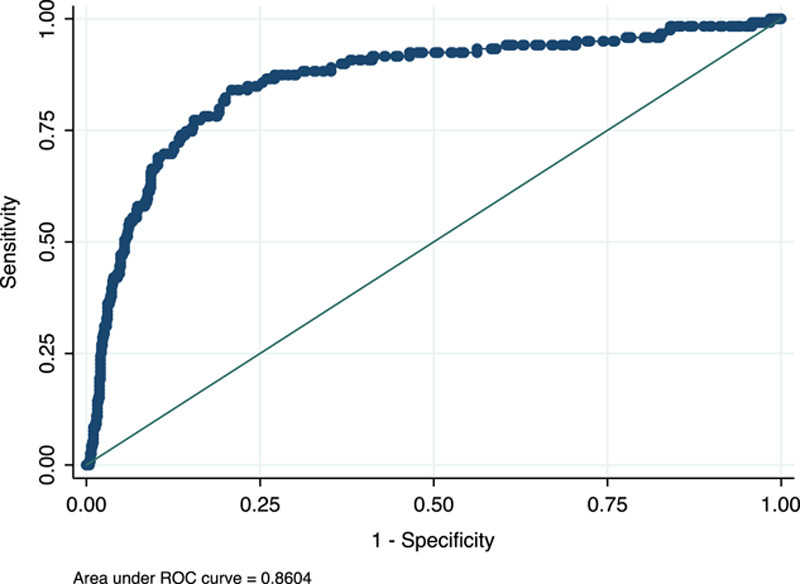
AUROC of POC dIgA ratio for portal hypertension (n = 809). AUROC = 0.86 (95% CI: 0.78–0.86, *p* < 0.001). Abbreviations: AUROC, area under the receiver operating characteristic curve; dIgA, dimeric IgA to monomeric IgA ratio; POC, point-of-care.

Within the subset of 809 patients in whom data on portal hypertension status was available, 119 (15%) had portal hypertension. AUROC of POC dIgA ratio for diagnosis of portal hypertension was 0.86 (0.78–0.86, *p* < 0.001; Figure 7). Using an optimized dIgA ratio cutoff of 0.6 yielded a sensitivity of 84%, specificity of 78%, PPV of 40%, and NPV of 97% for portal hypertension. A further subset of 688 patients had data on recent endoscopy to confirm the presence or absence of esophageal varices; among this group, the POC dIgA ratio (cutoff of 0.6) had 71% sensitivity, 80% specificity, and 99% NPV for esophageal varices, however, the PPV was low at 10%. “Importantly, 92% of those with clinically significant portal hypertension detected by POC dIgA ratio did not have fluid overload or ascites, jaundice, encephalopathy, or other peripheral stigmata of cirrhosis and portal hypertension, therefore would not have been detected by physical examination.”

## DISCUSSION

This pilot study shows that plasma dIgA ratio is elevated in cirrhosis and that a novel POC test based on dIgA ratio measurement in plasma has moderate accuracy for cirrhosis diagnosis, potentially overcoming a critical barrier to cirrhosis diagnosis and viral hepatitis treatment in low-resource and remote settings. Diagnosis of cirrhosis is an essential step in the management of all liver diseases to guide the implementation of preventive measures to reduce deaths from liver failure and liver cancer.^4^

We showed that the plasma dIgA ratio was elevated in people with liver cirrhosis compared with those without cirrhosis, irrespective of underlying liver disease etiology. Importantly, a dose-response association was observed: dIgA ratio levels increased with increasing disease severity and correlated markers of liver function (albumin and bilirubin). In addition, we found that the dIgA ratio was significantly elevated in patients with portal hypertension, ascites, and hepatic encephalopathy, which are all associated with a state of gut leakage; this supports but does not prove our hypothesis that the dIgA ratio is elevated in states of gut leakage.

Studies have previously shown a positive association between plasma dIgA levels and cirrhosis, however, these were exclusively in patients with alcohol-associated cirrhosis,^20^ whereas in this study most patients had viral hepatitis. Chronic HBV infection is associated with more insidious inflammation and immune exhaustion.^26^ Moreover, in our cohort, most patients with HBV had no cirrhosis. These factors might explain why dIgA performed better as a marker for cirrhosis due to HCV and other causes than for HBV. Further studies of the mechanisms of dimeric IgA elevation in cirrhosis are needed to determine the significance of these findings. Importantly, we found that dIgA ratio results were stable over time for cirrhosis diagnosis: only 3% of sample pairs had significantly discordant results, one third of which occurred in the setting of an alanine aminotransferase flare, which may conceivably be associated with higher gut leakage.

Using a dual cutoff POC dIgA ratio as a screening test for cirrhosis, followed by further investigation for those with an indeterminate POC dIgA ratio result identified 79% of all patients with cirrhosis, an improvement on the 55% sensitivity offered by APRI alone. Despite the modest PPV and the dual cutoff approach requiring 43% of people in our cohort to undergo further investigations for cirrhosis such as elastography, this approach provided same-day cirrhosis result within 30 minutes and avoided further investigations in 57% of the cohort. Moreover, the POC dIgA test is cheap to produce, easy to use with limited training, and could be provided in community settings that currently have no access to elastography or liver biopsy, where access to routine blood tests required for APRI and FIB-4 calculation is hampered by significant logistical, access and out-of-pocket cost barriers. Given the global prevalence of liver disease and current limited access to cirrhosis diagnostics in many low-resource and remote settings, a POC test for cirrhosis that removes the need for further testing in over half the population screened may still achieve a great impact on liver disease outcomes by increasing access to timely diagnosis and treatment for people with cirrhosis. Further optimization of the POC dIgA test and disease-specific cutoffs may improve test accuracy and reduce the proportion of test results in the intermediate zone that require further testing to confirm cirrhosis.

Importantly, the POC dIgA test also had good diagnostic accuracy for portal hypertension. Portal hypertension is associated with many of the life-threatening complications of liver cirrhosis, including acute variceal hemorrhage, ascites, spontaneous bacterial peritonitis, and hepatic encephalopathy.^4^ Treatment of portal hypertension with a nonselective generic β-blocker such as propranolol in the absence of significant renal impairment or acute liver decompensation improves survival and reduces the risk of variceal hemorrhage.^9^ The current gold standard for portal hypertension diagnosis is a gastroscopy to confirm the presence of portal hypertensive gastropathy and/or esophageal or gastric varices. Platelet count and elastography can also be used together to triage the risk of significant portal hypertension,^27^, and liver ultrasound may show evidence of an enlarged portal vein with reversal of flow, recanalization of collateral veins, and an enlarged spleen in portal hypertension. However, in many countries endoscopy access is very limited outside of large urban centers, and most people with cirrhosis in low-resource and remote settings do not have access to these tests.^11^ Importantly, the POC dIgA ratio detected clinically silent portal hypertension. This new assay may represent a simple low-cost measure to reduce death from portal hypertension in resource-constrained and/or remote settings.

POC diagnostic tests have been shown to be feasible and acceptable for population screening, particularly for hard-to-reach marginalized populations such as people who inject drugs,^28^ or in remote and resource-limited settings such as sub-Saharan Africa.^29^ They are widely used for the diagnosis of HBV and HCV and are recommended in World Health Organization (WHO) guidelines.^30^,^31^ POC diagnostic tests for HBV and HCV have been shown to improve linkage to care and reduce time to treatment through the provision of an on-the-spot result, facilitating decentralized models of care.^14^,^29^,^32^ To our knowledge, the POC dIgA ratio test is the first POC test for cirrhosis diagnosis and could potentially improve access to cirrhosis diagnosis in low-resource and remote settings. Further work is underway to adapt the POC dIgA ratio test for use with and with an optimized cutoff for whole blood and to provide a visual read, semiquantitative result, which would simplify the test for use in the field by trained health workers and avoid the need for use with the Axxin handheld reader.

Dimeric to monomeric IgA ratio was first noted to be elevated in liver cirrhosis decades ago.^20^ Previously, the detection of the small fraction of dimeric IgA in plasma involved the use of complex techniques such gel filtration chromatography or density-gradient ultracentrifugation.^33^ We have developed a novel reagent that binds selectively to dIgA, enabling measurement of dIgA by a lateral flow POC test device. In contrast, other described biomarkers for cirrhosis such as APRI^13^ require composite algorithms of multiple parameters which are less suitable for a single output POC test cartridge format. Our POC test based on the dIgA ratio measures a single blood parameter in a dual well device that is easy to use with minimal training at the POC. It provides a quantitative result in standardized Axxin units, thereby overcoming the costs and resourcing required to perform multiple blood test assays and the need to synchronize different laboratory assay ranges that are not always comparable. Moreover, the quantitative result format provides the capacity to optimize cutoffs for different populations and adapt to changing requirements for fibrosis assessment in management guidelines.

The underlying mechanisms of elevation of dimeric to monomeric IgA ratio in cirrhosis are not known and require further investigation. There are several possible explanations, including increased passive transfer of dIgA across the blood-gut barrier with reduced oncotic pressure, reduced hepatic clearance from plasma, or increased production and secretion into the blood by enterocytes or hepatocytes in response to greater bacterial translocation across the gut wall in states of gut leakage.^16^,^34^ Our work does not answer this question and further studies are needed to elucidate the underlying mechanisms of dimeric IgA elevation in cirrhosis. However, our translational work demonstrates a strong relationship between the dIgA ratio and portal hypertension, suggesting that gut leakage may be one driver of elevated plasma dIgA levels, rather than liver dysfunction and reduced plasma clearance.

Key strengths of this pilot study are the large sample size and the inclusion of diverse liver diseases within the cohort. However, there are several limitations. We did not have liver biopsy confirmation of cirrhosis in all patients; though transient elastography is considered the clinical standard for diagnosis of cirrhosis, liver biopsy histopathology would strengthen the classification of cirrhosis cases and would be an important inclusion in future study design. Similarly, not all patients had transient elastography results available, meaning we could not determine the correlation between the dIgA ratio and Fibroscan liver stiffness measurements with confidence. Some patients may also have been on treatment for portal hypertension with β-blockers, which may have led to an underestimation of the association between the dIgA ratio and portal hypertension. dIgA ratio increased with increasing severity of liver dysfunction, particularly the presence of portal hypertension. This means that the POC dIgA test had lower sensitivity for those with occult, well-compensated cirrhosis, who are the most difficult group to detect clinically. While detection of portal hypertension is vital to reduce deaths from complications such as variceal bleeding and ascites, detection of early compensated Child-Pugh A cirrhosis is key to inform treatment decisions in HBV and identify patients who are at greater risk of cirrhosis complications. Our study showed the POC dIgA ratio test could detect clinically silent cirrhosis with an NPV of 90%, which was improved by restricting test use to those aged above 30 years to increase the pretest probability of cirrhosis. Notably, the POC dIgA test identified more patients with cirrhosis than APRI, who are the most likely to benefit from treatment to prevent life-threatening complications.

Further studies are needed to determine whether disease-specific cutoffs should be used to improve test accuracy for cirrhosis, particularly for alcohol-associated liver disease and metabolic-associated fatty liver disease, which were underrepresented in our cohort and may have differing levels of gut leakage to viral hepatitis. Cost-effectiveness modeling comparing the use of POC dIgA test to screen for cirrhosis with the standard of care is required to refine settings where the POC dIgA test is likely to have the greatest impact on improving cirrhosis diagnosis, increasing timely viral hepatitis treatment uptake and reducing liver-related deaths.

## CONCLUSIONS

The dimeric IgA ratio is a promising novel biomarker of liver cirrhosis, particularly among people with portal hypertension. The POC dIgA ratio test is cheap to produce, easy to use, and provides cirrhosis risk assessment at the POC. POC dIgA had good discriminative accuracy for detecting cirrhosis, with comparable sensitivity to APRI. Further studies validating the accuracy of the POC dIgA test for cirrhosis in other liver diseases are warranted.

## Supplementary Material
