# Supplementary material for: A novel point-of-care test for cirrhosis based on dimeric to monomeric IgA ratio in blood: a pilot cohort study
Source: Hepatol Commun. 2023 Mar 30;7(4):e0106. doi: 10.1097/HC9.0000000000000106 (PMC10069834; doi:10.1097/HC9.0000000000000106)
Supplement: Supplementary file 1 [file hc9-7-e0106-s001.docx]

**Supplementary Methods**

**Table S1. Distribution of clinical samples from contributing sources**

| **Source** | **Number samples** | **Number individuals** | **Number healthy controls** | **Number cirrhosis** | **Number HBV** | **Number HCV** |
| --- | --- | --- | --- | --- | --- | --- |
| TAP study | 295 | 168 | 0 | 3 | 0 | 168 |
| Austin Pre- Liver transplant biobank | 124 | 124 | 0 | 121 | 13 | 38 |
| St Vincent’s Hospital hepatitis B biobank | 74 | 74 | 0 | 17 | 74 | 0 |
| STOP stud | 300 | 61 | 0 | 01 | 61 | 0 |
| Royal Prince Alfred biobank | 84 | 84 | 0 | 84 | 8 | 32 |
| CATCH study | 622 | 355 | 0 | 1 | 355 | 0 |
| Burnet Institute Biobank | 16 | 16 | 16 | 0 | 0 | 0 |
| Burnet COVID19 biobank | 21 | 21 | 21 | 0 | 0 | 0 |
| St Vincent’s Hospital Liver Cancer biobank | 75 | 57 | 0 | 53 | 4 | 26 |
| Total | 1611 | 960 | 37 | 280 | 515 | 263 |

*HBV, hepatitis B; HCV, hepatitis C.*

**Table S2. Liver stiffness median (LSM) cutoffs for cirrhosis by aetiology(1)**

| **Aetiology** | **Cut-off LSM (kPa)** |
| --- | --- |
| Chronic hepatitis C | >12.5 |
| Chronic hepatitis B | >12 |
| Alcohol related liver disease | >23 |
| Non-alcohol related fatty liver disease | >12 |
| Other | >12.5 |

**Supplementary Results**

**Normal range reference, Inter-observer variability and coefficient of variance for the POC dIgA test**

A normal range reference for the study was determined using 37 healthy controls. In this group, median dIgA ratio was 0.39 (IQR 0.31, 0.51), with a range from 0.13 to 0.98. 90% of healthy controls had dIgA ratio values below 0.6.

A comparison of 5 duplicate samples from one patient with cirrhosis and one healthy control were compared to determine inter-observer variability and coefficient of variance for the POC dIgA test (**Supplementary Table S3**). The POC dIgA test had a standard deviation of 0.04 and coefficient of variance of 17.2 for the health control sample and 0.16 and 8.4 for the cirrhosis sample, respectively. All sample duplicates were below the cutoff for cirrhosis (0.6) in the healthy control sample and above the cutoff for the cirrhosis sample. A comparison between dIgA ratio measured in plasma compared with whole blood in duplicate samples from a patient with cirrhosis showed dIgA ratio results were in the cirrhosis range, however results were two-fold higher in whole blood than in plasma (**Supplementary Table S4**).

**Table S3. Inter-test variability and coefficient of variance: cirrhosis and healthy control**

| **Sample test number** | **Control** | **Monomeric IgA** | **Dimeric IgA** | **dIgA ratio** | **Cirrhosis range > 0.6** |
| --- | --- | --- | --- | --- | --- |
| **Cirrhosis sample** |  |  |  |  |  |
| Duplicate 1 | 3080 | 5920 | 9980 | 1.69 | Yes |
| Duplicate 2 | 3820 | 5930 | 13020 | 2.20 | Yes |
| Duplicate 3 | 4440 | 6320 | 12340 | 1.95 | Yes |
| Duplicate 4 | 4090 | 6270 | 12010 | 1.92 | Yes |
| Duplicate 5 | 3960 | 6160 | 12200 | 1.98 | Yes |
| Mean +/- sd | - | 6120 +/- 167 | 11910 +/- 1023 | 1.95 +/- 0.16 |  |
| Coefficient of variance | - | 2.74 | 8.59 | 8.35 |  |
| **Healthy Control sample** |  |  |  |  |  |
| Duplicate 1 | 2650 | 4680 | 1030 | 0.22 | No |
| Duplicate 2 | 1700 | 5420 | 780 | 0.14 | No |
| Duplicate 3 | 2290 | 4150 | 990 | 0.24 | No |
| Duplicate 4 | 2100 | 5340 | 980 | 0.18 | No |
| Duplicate 5 | 2450 | 4840 | 1110 | 0.23 | No |
| Mean +/- sd | - | 4886 +/- 464 | 978 +/- 109 | 0.20 +/- 0.04 | No |
| Coefficient of variance | - | 9.50 | 11.15 | 17.23 |  |

**Table S4. Comparison of POC test measurement of dIgA ratio in plasma and whole blood in duplicate samples from one person with cirrhosis (shown in duplicate)**

| **Sample** | **Control** | **mIgA** | **dIgA** | **dIgA ratio** | **Cirrhosis range > 0.6** |
| --- | --- | --- | --- | --- | --- |
| 1.Plasma | 3630 | 4740 | 9260 | 1.95 | Yes |
| 1.Blood | 1960 | 2220 | 8890 | 4.00 | Yes |
| 2.Plasma | 2830 | 4580 | 8680 | 1.90 | Yes |
| 2.Blood | 2300 | 1790 | 8620 | 4.82 | Yes |

**Table S5. Sensitivity and specificity of different POC dimeric IgA ratio cutoffs for liver cirrhosis: Test cohort** (n=260)

| Test cohort | | | | | | |
| --- | --- | --- | --- | --- | --- | --- |
| POC dIgA Ratio cutoff | Sensitivity | Specificity | PPV | NPV | AUROC | 95% CI |
| 0.40 | 85% | 61% | 53% | 89% | 0.68 | 0.66-0.79 |
| 0.50 | 82% | 77% | 64% | 89% | 0.79 | 0.74-0.84 |
| 0.60 | 74% | 86% | 75% | 87% | 0.80 | 0.75-0.85 |
| 0.70 | 65% | 92% | 80% | 84% | 0.78 | 0.73-0.84 |
| 1.0 | 46% | 99% | 94% | 81% | 0.73 | 0.66-0.79 |

To explore stability in test results over time, we compared 309 sample pairs taken 12 months apart. The difference in paired measures was not statistically significant (p=0.09). Of the 309 paired samples, 21 (7%) had dIgA ratio results that were discordant for cirrhosis status (defined by dIgA ratio cutoff of 0.6); only 8 (3%) were discordant by > 0.1. Of these 8 sample pairs, all were from non-cirrhotic HBV patients. Three pairs had very high ALT levels when a discordant cirrhosis range dIgA ratio result was measured; in these patients, subsequent POC dIgA testing was concordant with the second dIgA ratio result.

**Figure S1. Comparison of dIgA ratio between people with and without cirrhosis, by liver disease aetiology**

1. Median dIgA ratio is higher in cirrhosis (0.60 (0.37, 0.89) compared with no cirrhosis (0.37 (0.26,0.52) in people with hepatitis B (ranksum p<0.001).
2. Median dIgA ratio is higher in cirrhosis (median dIgA ratio 0.76 (0.45, 1.25) compared with no cirrhosis (0.40 (0.25, 0.54) in people with hepatitis C (ranksum p<0.001)
3. Median dIgA ratio is higher in cirrhosis (0.96 (0.58, 1.51) compared with no cirrhosis (0.40 (0.32, 0.52) in people with non-viral hepatitis liver disease (ranksum <0.001).

**Figure S2. Correlation between platelet count and dimeric IgA ratio**

Adj R^2^=-0.18, p<0.001.
